# Cracks in the Diagnosis: A Case of Hypermobility Spectrum Disorder as a Mechanical Mimic of Spondyloarthritis With a Brief Review

**DOI:** 10.7759/cureus.112152

**Published:** 2026-07-06

**Authors:** Syed Ahmad Moosa, Abira A Chowdhury, Eugeniya Golub, Aleksander Feoktistov

**Affiliations:** 1 Rheumatology, New York City (NYC) Health + Hospitals/Kings County and State University of New York (SUNY) Downstate Medical Center, New York, USA; 2 Internal Medicine, University at Buffalo, State University of New York, Buffalo, USA

**Keywords:** bone marrow edema, diagnostic mimic, generalized joint hypermobility, hypermobility spectrum disorder, mechanical back pain, sacroiliac joint mri, sacroiliitis, spondyloarthritis

## Abstract

Adolescents commonly present with chronic back and knee pain, and distinguishing mechanical from inflammatory causes is a frequent challenge in general and pediatric practice. Juvenile spondyloarthritis (jSpA) is a chronic inflammatory disease affecting joints, entheses, and potentially the axial skeleton that may require prolonged immunosuppression to prevent structural joint damage. Generalized joint hypermobility (GJH) and hypermobility spectrum disorder (HSD) can present with overlapping musculoskeletal symptoms, including chronic joint pain and back stiffness, and may therefore be mistaken for inflammatory disease. Sacroiliac joint bone marrow edema on magnetic resonance imaging (MRI), although an important feature of axial spondyloarthritis, may also be seen in healthy individuals, athletes, postpartum women, and mechanically stressed joints, particularly when interpreted without adequate clinical correlation. This case report and brief review aim to illustrate how GJH can mimic jSpA and how overinterpretation of sacroiliac MRI findings may lead to unnecessary immunosuppression in adolescents.

A 17-year-old male from Bangladesh presented for an independent pediatric rheumatology evaluation after a prior diagnosis of jSpA based on reported sacroiliac MRI abnormalities, partial response to nonsteroidal anti-inflammatory drugs (NSAIDs), and intermittent gastrointestinal symptoms. His inflammatory markers were persistently normal, HLA-B27 testing was negative, and his back pain lacked inflammatory features. Examination demonstrated GJH with a Beighton score of 7/9. Repeat independent sacroiliac MRI was normal, and knee MRI showed a partial anterior cruciate ligament injury with bone contusion, supporting a mechanical etiology. Genetic testing for heritable connective tissue disorders was negative.

Expert consultation, including review by specialists affiliated with the Ehlers-Danlos Society and an independent pediatric rheumatologist at the National University Health System in Singapore, favored generalized HSD over jSpA. Structured physiotherapy and conservative rehabilitation led to complete symptom resolution without immunosuppressive therapy.

This case highlights the risk of overdiagnosing spondyloarthritis when nonspecific sacroiliac MRI findings are prioritized over the clinical pattern, laboratory data, and mechanical risk factors. GJH should be systematically assessed in adolescents with chronic musculoskeletal pain, especially when inflammatory markers are normal and symptoms are predominantly activity-related.

## Introduction

Sacroiliitis is defined as inflammation of one or both sacroiliac (SI) joints, which form the mechanical bridge between the lower spine and pelvis. It is a hallmark imaging feature of juvenile-onset spondyloarthritis (jSpA), a group of inflammatory disorders characterized by arthritis, enthesitis, and, in some patients, axial involvement [1]. Pathologically, inflammatory sacroiliitis may involve synovitis and enthesitis and may progress to structural damage, including erosions, sclerosis, and ankylosis.

SI joint abnormalities on imaging may be inflammatory or noninflammatory. Inflammatory sacroiliitis is classically associated with HLA-B27 and axial spondyloarthritis. In contrast, noninflammatory causes include mechanical or degenerative conditions, such as osteitis condensans ilii, degenerative osteoarthritis, and anatomic variants such as accessory SI joints [2,3]. High-impact physical activity or ligamentous laxity, as seen in hypermobility spectrum disorder (HSD), can generate mechanical shearing forces across the SI joint and may produce bone marrow edema (BME) that mimics inflammatory disease on MRI [4,5].

MRI is highly sensitive for detecting active inflammation through BME on short tau inversion recovery (STIR) or other fluid-sensitive sequences, but its specificity is limited when imaging findings are interpreted in isolation [1,3]. SI joint BME meeting the Assessment of SpondyloArthritis international Society (ASAS) definition for sacroiliitis has been reported in healthy asymptomatic individuals and is more frequent in populations exposed to mechanical stress, including athletes and postpartum women [6].

In patients with generalized joint hypermobility (GJH), ligamentous laxity may predispose them to repetitive microtrauma, mechanical instability, transient joint effusions, and reactive marrow signal changes. These features can create diagnostic confusion and may lead to unnecessary long-term immunosuppression if the clinical pattern is not carefully integrated with laboratory and imaging data [7,8]. We present the case of a 17-year-old male from Bangladesh who was initially diagnosed with Juvenile spondyloarthritis (jSpA) and treated with immunosuppression on the basis of nonspecific MRI findings and chronic musculoskeletal pain but was ultimately diagnosed with generalized HSD after multidisciplinary review.

## Case presentation

Initial presentation and diagnostic history

A 17-year-old male from Bangladesh, born to consanguineous parents who were first cousins, sought an independent virtual (remote) pediatric rheumatology evaluation in June 2025. His symptoms began at age 15 after a basketball-related injury to the left knee. The injury was initially managed as a mechanical problem, but persistent knee pain and subsequent intermittent low back discomfort prompted further evaluation in his home country. One year later, in November 2024, he sustained a second sports-related injury involving the right knee.

The patient also reported intermittent abdominal discomfort and occasional loose bowel movements. He denied fever, constitutional symptoms, rash, psoriasis, oral ulcers, or ocular complaints.

Initial SI joint radiographs were reported to show mild bilateral marginal sclerosis with blurring of the articular margins, along with left-sided hemisacralization of L5 (fifth lumbar vertebra). MRI of the SI joints was subsequently interpreted as showing mild bilateral acute sacroiliitis on a background of chronic sacroiliitis, greater on the right than the left. Based on the reported grade I sacroiliitis, intermittent gastrointestinal symptoms, and partial response to nonsteroidal anti-inflammatory drugs (NSAIDs), he was diagnosed with JSpA with peripheral involvement by an outside rheumatologist and was started on sulfasalazine approximately six months before the independent evaluation.

Clinical evaluation and physical examination

At the time of reassessment, the patient's musculoskeletal symptoms were more consistent with a mechanical pattern than an inflammatory one. Knee pain worsened with activity and weight-bearing and improved significantly with rest. He described episodic knee instability and brief morning stiffness lasting only 5-10 minutes. His back discomfort was intermittent and improved partially with NSAIDs and self-directed back-cracking maneuvers. He also reported intermittent diarrhea and abdominal discomfort but denied dysuria, urethral discharge, genital ulcers, or other symptoms suggestive of a genitourinary infection or sexually transmitted infection. He also denied recurrent syncope, presyncope, palpitations, orthostatic intolerance, flushing, recurrent urticaria, angioedema, or anaphylaxis-like episodes. Review of systems was negative for hematochezia, nocturnal gastrointestinal symptoms, weight loss, uveitis, psoriasis, or other spondyloarthritis (SpA)-defining extra-articular features. Family history was notable for GJH in the father, who had similar joint laxity but had not undergone formal evaluation.

**Figure 1:**
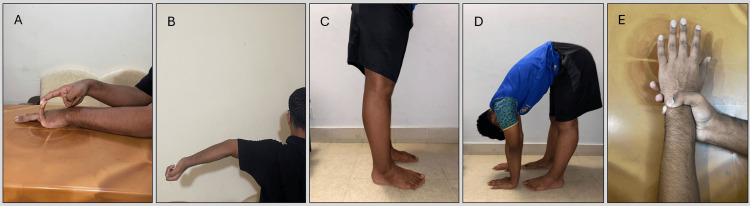
Clinical photographs demonstrating generalized joint hypermobility (GJH). (A) Passive dorsiflexion of the fifth metacarpophalangeal joint beyond 90 degrees, demonstrated with the hand resting on a flat surface; (B) elbow hyperextension beyond 10 degrees with the arm extended; (C) knee hyperextension (genu recurvatum) beyond 10 degrees in the standing position; (D) forward flexion with both palms placed flat on the floor while maintaining fully extended knees, demonstrating increased spinal and hamstring flexibility; and (E) positive wrist sign (Walker-Murdoch sign), with overlap of the thumb and fifth finger around the contralateral wrist. Features shown in panels A-D supported GJH, with a Beighton score of 7/9, while the positive wrist sign (Walker-Murdoch sign) serves as a physical indicator of arachnodactyly (abnormally long, slender fingers) and joint hypermobility.

Virtual physical examination demonstrated marked GJH, including bilateral fifth-finger hyperextension, elbow and knee hyperextension, and increased spinal flexion. These findings corresponded to a Beighton score of 7/9 (Figure 1, Table 1) [9]. 

**Table 1:** Beighton scoring system for generalized joint hypermobility (GJH). This is a nine-point clinical scoring system used to assess GJH. Four maneuvers are assessed bilaterally, and one maneuver is assessed once. Higher scores indicate greater GJH. The 2017 international classification for hypermobile Ehlers-Danlos syndrome defines GJH using age-specific cutoffs: ≥6/9 for prepubertal children and adolescents, ≥5/9 for pubertal men and women up to age 50 years, and ≥4/9 for adults over age 50 years.

Maneuver	Scoring	Maximum points
Passive dorsiflexion of the fifth metacarpophalangeal joint beyond 90°	1 point per hand	2
Passive apposition of the thumb to the flexor aspect of the forearm	1 point per hand	2
Hyperextension of the elbow beyond 10°	1 point per elbow	2
Hyperextension of the knee beyond 10°	1 point per knee	2
Forward flexion of the trunk with knees fully extended and palms resting flat on the floor	1 point	1
Total		9

Prior laboratory and imaging evaluation

Available laboratory evaluation performed by the previous rheumatologist repeatedly showed normal inflammatory markers, including erythrocyte sedimentation rate (ESR) and C-reactive protein (CRP). HLA-B27 testing was negative. Anti-dsDNA and ferritin results were also unremarkable, as summarized in Table 2. 

**Table 2:** Serial laboratory investigations during diagnostic evaluation. Serial laboratory testing showed persistently normal inflammatory markers, including erythrocyte sedimentation rate and C-reactive protein, along with negative HLA-B27 testing, findings that argued against active inflammatory spondyloarthritis in the appropriate clinical context. ALT, alanine aminotransferase; anti-dsDNA, anti-double-stranded DNA antibody; ASO, antistreptolysin O; CRP, C-reactive protein; ESR, erythrocyte sedimentation rate; HLA-B27, human leukocyte antigen B27; MCV, mean corpuscular volume; WBC, white blood cell count.

Test	Reference range	Nov 2023	Jan 2024	Feb 2025	Apr 2025
Hemoglobin	13-17 g/dL	13.4	13.6	12.3	-
MCV	80-96 fL	86.5	-	-	-
WBC	4,000-11,000/µL	7,400	7,510	8,810	-
ESR	<20 mm/hr	7	5	8	5
CRP	<1.0 mg/dL	-	-	-	0.3
Creatinine	0.6-1.3 mg/dL	1.0	1.0	1.2	-
ALT	<63 U/L	10	20	21	-
Anti-dsDNA	<6 IU/mL	1.1	3.6	5.8	-
Ferritin	~20-300 ng/mL	-	-	-	49.53
ASO titer	<200 IU/mL	-	-	-	200
HLA-B27	Negative	-	Negative	-	Negative

As discussed above, radiographic reports described mild bilateral SI joint sclerosis suggestive of grade I sacroiliitis, while MRI was reported to show mild bilateral sacroiliitis with small areas of STIR hyperintensity, greater on the right than the left, without ankylosis or other structural damage. The original imaging studies corresponding to these reports were not available for independent review by the authors.

Diagnostic reassessment

The prior diagnosis of jSpA appeared to have been based primarily on three considerations: reported mild sacroiliitis on imaging, partial symptom improvement with NSAIDs, and intermittent gastrointestinal symptoms raising concern for possible inflammatory bowel disease-associated spondyloarthritis. However, several aspects of the presentation were atypical for inflammatory spondyloarthritis. The patient's back pain lacked classic inflammatory features, inflammatory markers were consistently normal, and HLA-B27 testing was negative. He also lacked uveitis, psoriasis, enthesitis, dactylitis, constitutional symptoms, and objective inflammatory arthritis.

In contrast, the patient demonstrated clear GJH, with a Beighton score of 7/9. His knee symptoms were more consistent with mechanical pain related to ligamentous injury and abnormal joint mechanics during sports, likely exacerbated by underlying ligamentous laxity. The initially reported SI joint edema was described as small and asymmetric, findings that may be seen in mechanically stressed joints and are not specific for inflammatory sacroiliitis. In addition, his intermittent gastrointestinal symptoms, in the absence of bleeding, nocturnal symptoms, or weight loss, were not strongly suggestive of inflammatory bowel disease.

Taken together, GJH provided a more unifying explanation for the patient's mechanical musculoskeletal symptoms than jSpA.

Further evaluation

**Figure 2:**
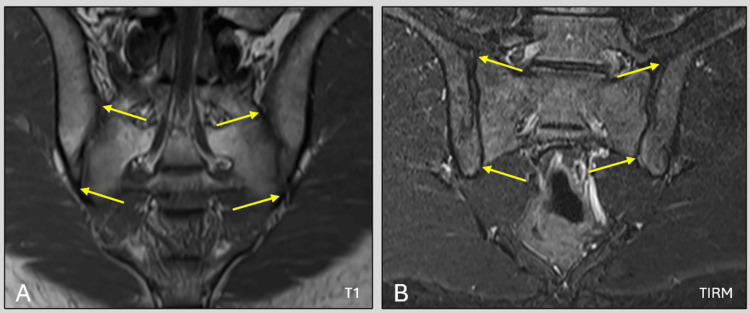
Coronal MRI images of the sacroiliac joints. A coronal T1-weighted image (A) and a nearly corresponding TIRM (turbo inversion recovery magnitude) fluid-sensitive image (B) show preserved sacroiliac joints without evident bone marrow edema or structural lesions. TIRM is a T2-weighted fat-suppressed sequence comparable to short tau inversion recovery (STIR). Yellow arrows delineate the extent of the bilateral SI joints and adjacent subchondral regions visible on these particular slices. Because accurate SI joint evaluation requires review of the complete multiplanar, multislice MRI series, these images are illustrative only; additional slices are available upon request.

Because the prior diagnosis of jSpA depended heavily on reported sacroiliitis, repeat imaging was obtained at an independent center. Repeat SI joint MRI demonstrated normal joint contours and no BME, erosions, sclerosis, ankylosis, or other features supporting active or structural sacroiliitis (Figure 2). Note that TIRM is a T2-weighted fat-suppressed sequence comparable to short tau inversion recovery (STIR), acquired using a turbo spin-echo readout; both suppress fat signal and highlight marrow edema, and either sequence may be used to assess active inflammatory sacroiliitis on MRI [3]. 

Knee MRI showed a partial anterior cruciate ligament (ACL) injury and bone contusion in the left knee, while the right knee MRI was normal. The imaging chronology is summarized in Table 3. 

**Table 3:** Complete imaging chronology. ACL, anterior cruciate ligament; L5, fifth lumbar vertebra; MRI, magnetic resonance imaging; SI, sacroiliac; STIR, short tau inversion recovery.

Study	Date	Findings
X-ray, right knee	Nov 2023	Normal
X-ray, SI joints	Nov 2023	Grade I bilateral sacroiliitis reported, with marginal sclerosis and blurring
Lumbar spine X-ray	Nov 2023	Hemisacralization of L5 on the left
MRI, SI joints	Jan 2024	Mild bilateral active sacroiliitis reported, with small STIR hyperintense areas, right greater than left; no ankylosis. Images were not available for review.
MRI, SI joints	Apr 2025	Completely normal: normal joint contours, normal marrow signal, no edema, erosion, or sclerosis. These images were available for review (see above).
MRI, right knee	Apr 2025	Normal
MRI, left knee	Apr 2025	Partial ACL tear, bone contusion of the lateral tibial condyle, and trace effusion

The differential diagnosis was revisited. The main diagnostic considerations and the clinical features supporting or arguing against each diagnosis are summarized in Table 4.

**Table 4:** Differential diagnosis considered during diagnostic reassessment. ACL, anterior cruciate ligament; ESR, erythrocyte sedimentation rate; CRP, C-reactive protein; hEDS, hypermobile Ehlers-Danlos syndrome; HLA-B27, human leukocyte antigen B27; MRI, magnetic resonance imaging; SI, sacroiliac.

Differential diagnosis	Supporting features	Features arguing against diagnosis
Juvenile spondyloarthritis	Intermittent low back stiffness; prior imaging reports suggestive of sacroiliitis; partial symptom improvement with NSAIDs; intermittent gastrointestinal symptoms	Back pain lacked classic inflammatory features; ESR/CRP remained normal; HLA-B27 was negative; no uveitis, psoriasis, enthesitis, dactylitis, or objective inflammatory arthritis; repeat SI joint MRI was normal
Inflammatory bowel disease-associated spondyloarthritis	Intermittent abdominal discomfort and loose bowel movements with musculoskeletal symptoms	No hematochezia, melena, nocturnal bowel movements, persistent diarrhea, or weight loss; gastrointestinal symptoms were mild/intermittent; inflammatory markers were normal
Reactive arthritis	Intermittent loose stools and lower-extremity musculoskeletal pain	No preceding acute infectious syndrome; no urinary symptoms, urethral discharge, genital ulcers, conjunctivitis, or mucocutaneous findings; chronic mechanical pattern; inflammatory markers were normal
Infectious sacroiliitis	Initial MRI report described asymmetric sacroiliac findings	No fever or constitutional symptoms; inflammatory markers were normal; symptoms were chronic rather than acute; repeat SI joint MRI was normal
Generalized hypermobility spectrum disorder	Beighton score 7/9; mechanical knee and back symptoms; recurrent sports-related injuries; family history of hypermobility; left knee MRI showing partial ACL injury and bone contusion	Most consistent diagnosis; provided a unifying explanation for the patient’s symptoms and mechanical imaging findings
Hypermobile Ehlers-Danlos syndrome	Generalized joint hypermobility, family history of hypermobility, and chronic musculoskeletal symptoms	Remained a clinical consideration; formal hEDS criteria could not be fully established from the available evaluation, and further connective tissue disorder assessment was pursued
Monogenic connective tissue disorder	Young age, marked hypermobility, and parental consanguinity	Considered because of consanguinity, father’s hypermobility, and patient’s marked hypermobility; no syndromic features such as severe skin fragility, vascular events, ocular abnormalities, major skeletal dysplasia, or early severe scoliosis were identified clinically, but genetic evaluation was pursued

Overall, the lack of objective inflammation on examination, unremarkable repeat SI MRI findings, and the presence of GJH with mechanical knee pathology made jSpA unlikely and favored generalized HSD as the working diagnosis. Evaluation for other heritable connective tissue disorders, such as Ehlers-Danlos syndrome and related disorders, was also pursued, given the finding of generalized hypermobility and parental consanguinity.

Initial management consisted of conservative therapy with referral to physical medicine and rehabilitation for strengthening and stabilization exercises, activity modification, joint protection, and bracing as needed. NSAIDs were recommended for pain control. The patient was counseled to remain on sulfasalazine, prescribed by the previous rheumatologist, in the interim, with the plan to discontinue it if the additional workup did not support inflammatory spondyloarthritis. He was also advised to follow up with gastroenterology for his GI symptoms if they persisted or worsened.

Genetics and expert evaluation

Next-generation sequencing using a comprehensive panel for Ehlers-Danlos syndrome and related connective tissue disorders was negative. This did not rule out HSD or hypermobile Ehlers-Danlos syndrome, since most patients with these diagnoses do not have an identifiable genetic variant on currently available testing.

The case was further reviewed with a recognized expert in heritable connective tissue disorders at Ghent University, Belgium, who is affiliated with the Ehlers-Danlos Society. Given the clinical presentation and negative genetic testing, a rare monogenic form of Ehlers-Danlos syndrome was considered unlikely. Overall, the findings were considered to be most consistent with HSD or hypermobile Ehlers-Danlos syndrome.

External rheumatologic assessment

Unfortunately, the treating rheumatologists in Bangladesh continued to support the diagnosis of jSpA despite the repeat imaging findings and advocated starting treatment with tofacitinib. They reportedly interpreted the repeat MRI as showing mild sacroiliitis; however, this contradicted the official radiology report, which noted normal SI joints. We also did not identify any sacroiliitis on our review of the MRI images.

Considering the diagnostic uncertainty at that point and the potential consequences of starting chronic immunosuppression in a teenager, along with the significant side-effect profile of the medication, we recommended an independent third-party rheumatology opinion based on an in-person evaluation. The patient was evaluated by a pediatric rheumatologist at the National University Health System in Singapore in June 2025, who confirmed that, after examination and review of the laboratory findings and imaging, there was no clinical or laboratory support for juvenile idiopathic arthritis (JIA) or jSpA. The final assessment was that his symptoms were most likely related to the mechanical components of musculoskeletal pain associated with GJH (supported by his history of joint hypermobility and fulfillment of the diagnostic criteria). Conservative management with physiotherapy, joint protection strategies, and muscle strengthening was recommended. Sulfasalazine was discontinued.

Outcome and follow-up

The patient was transitioned off immunosuppression and onto a physical and occupational therapy program focused on intensive strengthening and joint stabilization after significant education of both the patient and his parents. He has made continued improvement with the resolution of his musculoskeletal symptoms on this treatment plan and remains on it today. The patient was functional at his baseline activity level by January 2026. No other immunosuppressant was restarted.

**Figure 3:**
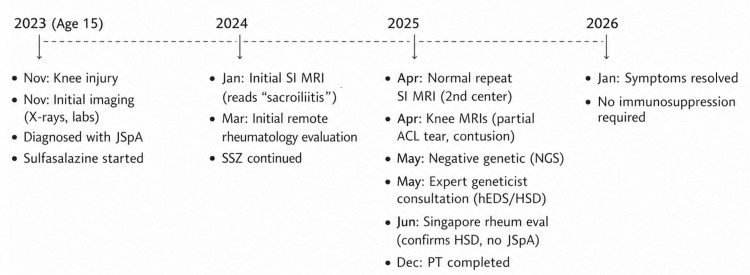
Timeline summarizing the patient’s diagnostic course from initial knee injury and reported sacroiliac imaging abnormalities to diagnostic reassessment, conservative management, and complete symptom resolution. SI, sacroiliac; MRI, magnetic resonance imaging; jSpA, juvenile spondyloarthritis; SSZ, sulfasalazine; ACL, anterior cruciate ligament; NGS, next-generation sequencing; hEDS, hypermobile Ehlers-Danlos syndrome; HSD, hypermobility spectrum disorder; PT, physical therapy.

The patient reported minimal gastrointestinal symptoms at this time and opted to forgo further evaluation by a gastroenterologist. He was advised to obtain an evaluation if symptoms returned, worsened, or developed alarm features (i.e., bleeding, nocturnal symptoms, or weight loss). To summarize the patient's clinical course, a timeline is shown below (Figure 3).

## Discussion

This case illustrates several pitfalls in evaluating adolescents with chronic musculoskeletal pain and imaging findings reported as sacroiliitis. The diagnostic trajectory, from an initial diagnosis of jSpA to reclassification as generalized HSD, emphasizes the importance of integrating the pain pattern, physical examination, laboratory data, imaging quality, and mechanical risk factors before committing a patient to long-term immunosuppression.

First, the ASAS classification criteria, originally developed in 2009, were recently revised in collaboration with the Spondyloarthritis Research and Treatment Network (SPARTAN) using the CLASSIC (CLassification of Axial SpondyloarthritiS Inception) inception cohort. Both the 2009 ASAS and the 2025 ASAS-SPARTAN criteria remain research classification tools, not diagnostic criteria for individual patients [1,10,11]. Overdiagnosis remains a practical concern; a recent Dutch survey found that 54% of rheumatologists used the ASAS classification criteria to diagnose non-radiographic axial spondyloarthritis (nr-axSpA) [12]. The SPACE (SPondyloArthritis Caught Early) cohort of 500 patients with suspected axial SpA (axSpA) also showed that SpA features increase diagnostic probability but are not determinative: the probability of axSpA increased with the number of SpA features, yet 17.4% of patients who fulfilled the ASAS classification criteria were not diagnosed with axSpA by the treating rheumatologist [13].

Second, the initial MRI finding of small STIR hyperintense areas was interpreted as active sacroiliitis, but BME is not specific for inflammatory disease. Overreliance on BME as the principal determinant of an ASAS-positive MRI may lead to overcalling inflammatory sacroiliitis in clinical practice [6,14]. Among healthy volunteers, 23.4% had MRI findings meeting the ASAS definition of sacroiliitis; among recreational runners and elite ice hockey players, 30%-41% met the same threshold, predominantly in the posterior lower ilium [5,6]. Data-driven thresholds, including at least four SI joint quadrants with BME or BME in at least three consecutive slices, have been proposed to improve specificity for active axial spondyloarthritis, with positive predictive values of at least 95% [15]. In this patient, the normal repeat MRI, absence of active or structural sacroiliitis, persistently normal inflammatory markers, and complete recovery with conservative management strongly supported a mechanical rather than an inflammatory explanation.

Third, GJH provided a unifying biomechanical explanation. Ligamentous laxity reduces passive stabilization of the SI joint, allowing excessive micromotion during weight-bearing and dynamic activity. This repetitive mechanical stress can produce subchondral microtrauma and reactive BME on STIR or other fluid-sensitive sequences, the same signal often used to define active sacroiliitis under the ASAS criteria [4,16]. The partial ACL tear and bone contusion on knee MRI further supported a sports-related mechanical phenotype rather than systemic inflammatory arthritis [17,18].

Another mechanical mimic is osteitis condensans ilii (OCI), a benign condition characterized by triangular sclerosis of the iliac bone with sacral sparing and joint-space preservation, most often described in young women after pregnancy [19]. Unlike inflammatory sacroiliitis, OCI typically lacks erosions, ankylosis, and inflammatory BME and is usually asymptomatic or associated with mechanical low back pain. Although OCI was not applicable to this patient, it reinforces the broader principle that mechanical stress can generate imaging abnormalities mistaken for inflammatory disease.

This case highlights many intersecting cognitive errors that predispose to diagnostic error in patients with suspected spondyloarthritis [20]. Diagnostic anchoring was likely present, as once sacroiliitis was initially reported, it became the lens through which the rest of the patient's features were filtered during the diagnostic workup. Diagnostic momentum likely followed once spondyloarthritis was diagnosed, as it became difficult to reconsider the diagnosis in the face of contradictory findings. Confirmation bias likely played a role, as the partial response to NSAIDs and intermittent gastrointestinal symptoms could have been overvalued as supporting evidence for SpA, while persistently normal inflammatory markers, negative HLA-B27, lack of enthesitis, uveitis, psoriasis, or objective inflammatory arthritis, normal repeat SI joint MRI, and mechanical findings on knee MRI were all strong arguments against an inflammatory process. Combined, these decision-making pitfalls may have led to consideration of escalation to tofacitinib despite evidence supporting a mechanical cause related to hypermobility [21].

The patient's intermittent abdominal discomfort and loose stools initially raised concern for inflammatory bowel disease-associated SpA. However, functional gastrointestinal symptoms are highly prevalent in hypermobility disorders and may offer a noninflammatory explanation for mild intermittent complaints. A large case-control study found that 98% of individuals with HSD/hEDS met the criteria for one or more Rome IV functional gastrointestinal disorders, with diarrhea reported in 47% compared with 9% of controls [22]. Proposed mechanisms include connective tissue laxity affecting gastrointestinal wall compliance, dysmotility, and autonomic dysfunction [23]. In this patient, the absence of alarm features, including hematochezia, nocturnal symptoms, and weight loss, along with spontaneous improvement, favored hypermobility-associated functional gastrointestinal symptoms rather than inflammatory bowel disease.

Parental consanguinity appropriately raised concern for an autosomal recessive EDS subtype, such as classical-like, kyphoscoliotic, or spondylodysplastic EDS. However, comprehensive genetic testing was negative for known EDS-associated genes. The father's hypermobility supported a familial hypermobility phenotype, consistent with hEDS/HSD, which is inherited in an autosomal dominant pattern with variable expression [24].

Lastly, this case highlights the importance of independent imaging review and a multidisciplinary second look. A repeat SI joint MRI obtained at a separate facility ultimately showed no BME, erosions, sclerosis, or other abnormalities. Inter-reader variability in SI joint MRI interpretation is well recognized, with studies showing meaningful disagreement between local and central readers and between radiologists and rheumatologists, particularly for subtle or borderline lesions [14,25]. In resource-limited settings, where access to experienced musculoskeletal radiologists may be constrained, a second independent imaging opinion before initiating or escalating long-term immunosuppression may be practice-changing. The patient's musculoskeletal symptoms improved with appropriate physical and occupational therapy, which is the cornerstone of conservative management of syndromic hypermobility [26].

Summary

This case underscores that the ASAS criteria are intended for classification rather than stand-alone diagnosis and that MRI BME is not specific for inflammatory sacroiliitis. Mechanical stress from hypermobility, athletic activity, pregnancy-related changes, or osteitis condensans ilii may mimic sacroiliitis. Therefore, generalized hypermobility, sports-related injuries, and family history should be carefully assessed, particularly in adolescents with mechanical back pain, normal inflammatory markers, and equivocal imaging. Intermittent gastrointestinal symptoms alone do not establish inflammatory bowel disease-associated spondyloarthritis. When findings remain uncertain, awareness of cognitive biases and repeat or independent imaging review may prevent misdiagnosis and unnecessary immunosuppression. In this patient, physiotherapy, joint protection, and activity modification resulted in complete recovery without further immunosuppressive treatment.

## Conclusions

This case illustrates how applying the ASAS classification criteria as diagnostic criteria, together with overinterpretation of nonspecific SI joint MRI changes, may contribute to a diagnosis of spondyloarthritis and subsequent exposure to potentially unnecessary immunosuppression. In this patient, the overall clinical course, including GJH, a mechanical pain distribution, persistently normal inflammatory markers, negative HLA-B27 testing, normal repeat SI joint MRI, mechanical knee pathology, and complete symptom resolution with rehabilitation, was more consistent with generalized HSD than JSpA.

As emphasized by the 2025 ASAS-SPARTAN criteria, these remain research classification criteria and should not be used as standalone diagnostic criteria. Diseases and conditions that mimic spondyloarthritis should be carefully considered, and imaging findings should be interpreted in the context of the full clinical picture. In adolescents with suspected spondyloarthritis, mechanical mimics such as hypermobility-associated stress changes and osteitis condensans ilii should be evaluated in the appropriate clinical context before initiating or escalating chronic immunosuppressive therapy.
